# A review of cyber vigilance tasks for network defense

**DOI:** 10.3389/fnrgo.2023.1104873

**Published:** 2023-04-18

**Authors:** Oliver Alfred Guidetti, Craig Speelman, Peter Bouhlas

**Affiliations:** ^1^Edith Cowan University, Joondalup, WA, Australia; ^2^Cyber Security Cooperative Research Centre, Perth, WA, Australia; ^3^Experimental Psychology Unit, Perth, WA, Australia; ^4^Western Australian Department of the Premier and Cabinet, Perth, WA, Australia

**Keywords:** vigilance, tasks, cyber defense, Security Event Information Management, vigilance decrement, sustained attention response task

## Abstract

The capacity to sustain attention to virtual threat landscapes has led cyber security to emerge as a new and novel domain for vigilance research. However, unlike classic domains, such as driving and air traffic control and baggage security, very few vigilance tasks exist for the cyber security domain. Four essential challenges that must be overcome in the development of a modern, validated cyber vigilance task are extracted from this review of existent platforms that can be found in the literature. Firstly, it can be difficult for researchers to access confidential cyber security systems and personnel. Secondly, network defense is vastly more complex and difficult to emulate than classic vigilance domains such as driving. Thirdly, there exists no single, common software console in cyber security that a cyber vigilance task could be based on. Finally, the rapid pace of technological evolution in network defense correspondingly means that cyber vigilance tasks can become obsolete just as quickly. Understanding these challenges is imperative in advancing human factors research in cyber security.

**CCS categories:**

Human-centered computing~Human computer interaction (HCI)~HCI design and evaluation methods.

## Introduction

The weakest link in modern network defense are the natural limitations of the human operators who work in security operations centers (Thomason, 2013; Cavelty, 2014). These limitations are neuropsychological in their origin, and mostly impact the human attentional system, which interacts with cognitive design elements of cyber security software. These elements of design include signal salience, event rate, cognitive load, and workload transitions (Parasuraman, 1979, 1985). The executive resources required to sustain vigilant attention to network defense systems are an order of magnitude greater than in classic vigilance domains, such as air traffic control, nuclear plant monitoring and baggage security (Wickens et al., 1997; Hancock and Hart, 2002; Chappelle et al., 2013; Gartenberg et al., 2015; Reinerman-Jones et al., 2016). The volume, diversity, specificity, and evolution rate of threats in the cyber landscape make network defense an extremely cognitively demanding task (D'Amico et al., 2005).

Classic vigilance research first involved creating a laboratory simulation of the operational sustained attention problem (Cunningham and Freeman, 1994; Smith, 2016; Joly et al., 2017; Valdez, 2019). For example, Mackworth's (1948, 1950) clock test was used to simulate the task demands associated with World War 2 radar operation. Because vigilance performance is task specific, the study of vigilance decrement in network defense analysts necessitates a test bed specifically designed to emulate the cognitive demands associated with real world cyber security (Satterfield et al., 2019). In this regard however, a gap has been identified in the tools available to investigate cyber vigilance decrement. Specifically, a validated cyber vigilance task that probes each of Parasuraman's (1979, 1985) parameters does not currently exist. This gap in the literature could hinder the application of wider human factors research, such as methods of tracking or intervening in vigilance decrement, from the lab into applied domains such as cyber security (Al-Shargie et al., 2019; Yahya et al., 2020). For example, Parasuraman's (1979, 1985) parameters of a valid vigilance tasks were derived long before modern network defense, it hence remains a similarly unexplored question if these parameters alone constitute a vigilance task valid in cyber security. Similarly, Bodala et al. (2016) demonstrated that integrating challenging features into vigilance task stimuli was a useful method of enhancing sustained attention. However, the task Bodala utilized was not designed to emulate the cognitive demands associated with modern cyber defense. Hence, it remains a standing question if the vigilance performance enhanced by greater challenge integration on Bodala's task would extend to cyber security. However, this question cannot be probed without a modern, validated cyber vigilance task in which the challenging parameters of stimuli can be controlled. The main goal of this review is therefore to understand several factors that may explain this gap in the literature, including access and confidentiality, task complexity, non-standard operating environments, and rapid obsolescence.

## Background

Situational awareness refers to the perception, comprehension, and projection of the threats within an environment across time and space (Endsley and Kiris, 1995; Wickens, 2008). The term cyber-cognitive situational awareness specifically refers to human operators' awareness of threats distributed across virtual landscapes (Gutzwiller et al., 2015). For the purposes of brevity, the term “cyber-cognitive situational awareness” is referred to here as “situational awareness.”

Network defense analysts must pay close consistent attention to Security Event Information Management Systems (SEIMs), which are used to establish and support situational awareness of cyber threat landscapes (Komlodi et al., 2004; Spathoulas and Katsikas, 2010, 2013; Tyworth et al., 2012; Albayati and Issac, 2015; Newcomb and Hammell, 2016). SEIMs summarize anomalous and potentially malicious patterns of network traffic as sets of alarms, or alerts, which analysts must individually investigate as potential cyber threats (Barford et al., 2010; Spathoulas and Katsikas, 2010, 2013; Gaw, 2014; Newcomb and Hammell, 2016). Analysts' capacity to sustain attention to their SEIM therefore constrains their situational awareness of the cyber threat landscape being protected (Endsley and Kiris, 1995; Gutzwiller et al., 2015; Wickens et al., 2015).

Situational awareness hinges on the capacity to sustain attention to threats distributed across cyber threat landscapes (Endsley and Kiris, 1995; Barford et al., 2010). In the context of network security, analysts use SEIMs to perceive and act on threats to protected cyber infrastructures (Gutzwiller et al., 2015). SEIM threat detection is a tedious, monotonous task that requires analysts to sustain high levels of attention for prolonged periods of time (Fathi et al., 2017; Nanay, 2018).

Distinguishing between malicious and benign SEIM alerts is not dissimilar to the search for a needle in a haystack (Erola et al., 2017). Analysts sift through vast numbers of SEIM alerts, most of which are false positives, just to identify and act on a small number of malicious threats (Sawyer et al., 2016). Although SEIM threat detection is initially easy to perform, analyst mistakes invariably accumulate with time spent distinguishing between malicious and benign element signals (Sawyer et al., 2016). This gradual decline in sustained attention is known as *vigilance decrement*; it occurs when the brain is required to sustain a high level of workload processing activity for longer than its energy reserves can support (Sawyer et al., 2016). Establishing and sustaining situational awareness in a cyber security operations center, requires that analysts sustain vigilant attention to their SEIM dashboards for prolonged periods of time (Wall and Williams, 2013). However, vigilance decrement has become an increasingly disruptive influence in operational network defense analysts whose role requires the use of SEIM to hunt for threats in the cyber landscape (Chappelle et al., 2013; Wall and Williams, 2013).

Vigilance refers to the capacity an individual has to sustain conscious processing of repetitive, unpredictable stimuli without habituation or distraction (Pradhapan et al., 2017). Vigilance is regarded as a state of alertness to rare and unpredictably frequent stimuli (Pradhapan et al., 2017). When attention is sustained for a prolonged period, human processing limitations lead to compounding performance failures, the phenomenon known as vigilance decrement (Sawyer and Hancock, 2018; Warm et al., 2018). For example, drivers must sustain vigilance in attuning and responding to hazards on the road (Zheng et al., 2019). A driver experiencing vigilance decrement, however, will be less capable of responding to road hazards (Gopalakrishnan, 2012). Hence, failure to sustain attention to road hazards is the leading cause of thousands of road deaths each year (Gopalakrishnan, 2012). Depending upon the context, vigilance decrement can manifest either as an increased reaction time to detect critical signals or as a reduction in their correct detection (Warm et al., 2018). For example, during World War Two, British radar operators were required to monitor their terminals over prolonged periods of time for “blips” that indicated the presence of Axis U-boats. Despite their training and motivation to avoid Axis invasion, these operators began to miss critical U-boat signals after only half an hour of monitoring (Mackworth, 1948, 1950). Mackworth (1948, 1950) was commissioned by the Royal Air Force to study the problem, in what would become seminal vigilance research.

Mackworth (1948, 1950) devised a “Clock Test” that simulated the Royal Air Force's radar displays. This comprised of a black pointer that traced along the circumference of a blank, featureless clock-type face in 0.3-inch increments per second. At random points during the task, the radar pointer would increment twice in a row as a way of simulating the detection of a *U*-boat. Mackworth (1948, 1950) tasked observers with detecting these double jumps by pressing a button when one was seen. Despite the clarity of Mackworth's (1948, 1950) target signals, correct detections declined by 10% in the first 30 min of the 2-h-long task. This gradual drop in correct signal detection was the first laboratory demonstration of vigilance decrement. The phenomenon has since been demonstrated as one of the most ubiquitous and consistently replicated findings in the vigilance literature (Baker, 1959; Mackworth, 1968; Sostek, 1978; Parasuraman and Mouloua, 1987; Dember et al., 1992; Warm and Dember, 1998; Pattyn et al., 2008; Epling et al., 2016).

Laboratory vigilance tasks require correctly identifying rare target stimuli in an array for a prolonged period (Daly et al., 2017). Vigilance decrement typically onsets within 15 min of sustained attention, however it has been reported in as little as 8 min under particularly demanding situations (Helton et al., 1999; St John et al., 2006).

Vigilance decrement has only recently received recognition in the human-factors literature, as a cyber incident risk factor (Chappelle et al., 2013; Mancuso et al., 2014). For example, network defense analysts who experience vigilance decrement will decline in their capacity to attune to, detect, and act against threats presented in a SEIM console (McIntire et al., 2013). Vigilance decrement is therefore a human factor bottleneck to the protective benefit of SEIM software. That is, the cyber protection offered by SEIM software is bottlenecked by the capacity of its operators to sustain vigilant attention to the information it presents. Managing vigilance decrement first necessitates a nuanced understanding of the factors which contribute to declines in sustained attention to network defense consoles (McIntire et al., 2013). This may explain why current attempts to manage vigilance decrement in the human factors literature have focused on developing unobtrusive psychophysiological monitoring methods for indicating when the capacity to sustain attention capacity begins to decline (McIntire et al., 2013; Mancuso et al., 2014; Sawyer et al., 2016). However, the psychophysiological correlates of cyber vigilance decrement may not be adequately understood without an experimental test bed that accurately simulates the cognitive demands associated with modern network defense (McIntire et al., 2013; Mancuso et al., 2015; Sawyer et al., 2016).

The review that follows identifies limitations in experimental platforms that could be used to conduct human-in-the-loop studies of cyber vigilance decrement, and challenges that need to be overcome to fill this gap. The only cyber vigilance tasks documented in the literature to date are owned by The United States Air Force and are outdated simulations of the demands associated with modern network defense (McIntire et al., 2013; Mancuso et al., 2015; Sawyer et al., 2016). Beyond researchers, an accessible experimental test bed for human-in-the-loop studies of cyber vigilance decrement could also provide utility to business, government, and militaries, by informing training, selection, and software development standards (Alhawari et al., 2012; Ormrod, 2014).

### Review significance

As reliance on global cyber networks continues to grow, the extent of the impact of their compromise will also increase (Ben-Asher and Gonzalez, 2015; Goutam, 2015). Ensuring the security of these systems hinges on the optimized performance of human network defenders (Thomason, 2013; Cavelty, 2014). Lapses in network defender attention therefore have the potential to cripple the cyber infrastructure being guarded (Thomason, 2013; Cavelty, 2014). This includes virtual and physical military assets, governmental assets, central banking networks, stock market infrastructure as well as national power and telecommunications grids (Gordon et al., 2011; Jolley, 2012; Saltzman, 2013; Ormrod, 2014; Hicks, 2015; Skopik et al., 2016; Rajan et al., 2017). The integrity of these assets hinges on measuring and mitigating neurocognitive inefficiencies in network defenders' capacity to sustain vigilant attention to cyber security command and control consoles (Maybury, 2012). Managing the risk associated with cyber vigilance decrement will enhance the defense of critical global cyber infrastructures (Maybury, 2012; Wall and Williams, 2013). However, cyber vigilance tasks that allow researchers to study the decrement in network defense are not currently accessible to researchers (Maybury, 2012; McIntire et al., 2013; Mancuso et al., 2015; Sawyer et al., 2016).

## Cyber vigilance decrement

In under 20 min, a fully trained, motivated, and experienced network defense analyst's capacity to identify threats in their SEIM can begin to decline (McIntire et al., 2013). From a technological perspective, this phenomenon, known as vigilance decrement, has arisen in the cyber domain due to the gradual rise in the volume, diversity and specificity of data that network analysts must process to identify and act upon threats (D'Amico et al., 2005).

Cyber vigilance decrement has emerged as a defining human factor of network security (Tian et al., 2004; Maybury, 2012; Aleem and Ryan Sprott, 2013; Wall and Williams, 2013; Franke and Brynielsson, 2014; Gutzwiller et al., 2015; Vieane et al., 2016). For example, prevalence denial attacks involve flooding the SEIM of a target network with huge volumes of innocuous, non-malicious signals designed to intentionally induce vigilance decrement in defense analysts (Vieane et al., 2016). Once in this less attentive state, bad actors can improve their chance of implementing a successful attack on the target network (Vieane et al., 2016). Vigilance decrement is therefore a cyber-cognitive security vulnerability which must be studied and managed like any other vulnerability in network defense (Tian et al., 2004; Aleem and Ryan Sprott, 2013; Wall and Williams, 2013; Vieane et al., 2016).

### Existing cyber vigilance tasks

Whilst Google Scholar is not a database, it was chosen as the driving methodology for this review for its capacity to broadly scan wide breadths of academic literature (Tong and Thomson, 2015). Studies were only included in this review if they presented a sustained attention task specifically designed to emulate the cognitive demands associated with operating a cyber security console, like the SEIM software that network defense analysts use to sustain situational awareness of virtual threat landscapes. This process yielded only three examples in the literature of an experimental test bed that researchers could use to study vigilance decrement in network defense (McIntire et al., 2013; Mancuso et al., 2015; Sawyer et al., 2016).

The Cyber Defense Task (CDT) that McIntire et al. (2013) presented was the formative example of a cyber vigilance task in the literature. Mancuso et al. (2015) and Sawyer et al. (2016) followed soon after with their presentation of the Mancuso Cyber Defense Task (MCDT and MCDT-II). The discussion that follows presents a critical review of the CDT and MCDT. For example, the validity of these tasks as simulations of the demands associated with network defense may have declined between now and when they were published due to evolving complexity in network defense (Gutzwiller et al., 2015). Rapid obsolescence of cyber vigilance tasks may also reflect the need to consider cyber-cognitive parameters of SEIM consoles which, according to Parasuraman (1979, 1985), influence the probability of vigilance decrement. Hence any research based on existent platforms may not generalize well beyond the lab, let alone beyond the context of military cyber defense for which they were designed.

#### McIntire's Cyber Defense Task (CDT)

McIntire et al.'s (2013) formative CDT aimed to psychophysiologically identify the onset of vigilance decrement in a laboratory cyber-defense task. Although successful in monitoring vigilance performance, several methodological issues make it difficult to generalize McIntire et al.'s (2013) results to operational cyber defense. For instance, McIntire et al.'s (2013) sample comprised 20 military and civilian cyber defenders who participated in four, 40-min trials of the CDT. It is possible that the civilian participants McIntire et al. (2013) sampled did not have the same motivations or stressors as the active duty subset of their sample (Finomore et al., 2009). This compromise was however understandable, as cyber defense analysts are a difficult population to sample from, and the task did not require prior cyber defense training (Zhong et al., 2003, 2015; Rajivan et al., 2013).

The CDT was designed to simulate the cognitive demands associated with modern network defense. It is not possible to completely appraise the CDT as a cyber vigilance task, as only a brief account of the software was documented in the literature (McIntire et al., 2013; Sherwood et al., 2016). In addition, McIntire et al. (2013) and Sherwood et al. (2016) are the only studies that have made use of the CDT, and both were sponsored by the United States Air Force Research Laboratory (AFRL). Though it cannot be confirmed, it is possible that the CDT has been retained for the AFRL's exclusive research use, which limits the degree of scientific enquiry that can be made into cyber vigilance decrement on this task.

As described in McIntire et al. (2013), the CDT involved two subtasks that participants concurrently completed during the cyber vigilance task. The CDT's textual component required the participant to monitor and report the presence of three suspicious IP addresses and port combinations (Figure 2 in McIntire et al., 2013). Participants had to memorize these IP addresses beforehand and press a button to indicate when one was observed. The second component of McIntire et al.'s (2013) CDT was graphical and presented concurrently with the first textual component. Participants were presented with a live graph of simulated network traffic, which they monitored in case a threshold value, indicated by a red horizontal line, was exceeded (Figure 2 in McIntire et al., 2013). Participants indicated when traffic exceeded this limit by pressing a button.

McIntire et al. (2013) observed vigilance decrement in CDT performance, which also correlated with a series of ocular parameters that they recorded using an eye tracker. Participants' blink frequency and duration, eye closure percentage, pupil diameter, eccentricity, and velocity were all recorded as they performed the CDT. These measurements all correlated with changes in CDT performance over time, a result which accorded with an abundance of studies on vigilance while driving (Thiffault and Bergeron, 2003a,b; Tan and Zhang, 2006; D'Orazio et al., 2007; Sommer and Golz, 2010; Jo et al., 2014; Aidman et al., 2015; Cabrall et al., 2016; Zheng et al., 2019).

#### Validity concerns with the CDT

It was unclear if the ocular changes that McIntire et al. (2013) correlated with time spent on the CDT would extend beyond this laboratory analog, which is not as cognitively demanding as network defense in the real-world (Donald, 2008; Reinerman-Jones et al., 2010; Chappelle et al., 2013; Hancock, 2013). The complexity of network defense could explain why existing cyber vigilance tasks are considered oversimplified (Rajivan et al., 2013; DoD, 2014; Gutzwiller et al., 2016; Rajivan and Cooke, 2017). For instance, eleven key service skills are required by the United States Department of Defense network defense analysts (DoD, 2014). These cores skills include cryptology, oversight and compliance, reporting, cyber security, computer science, network exploitation, and technology operations (DoD, 2014). A case could be made that the CDT did require the use of reporting oversight and compliance, however eight of the 11 core skills were not built into McIntire et al.'s (2013) task. In contrast, Mackworth's (1948, 1950) clock test accurately simulated every feature of the radar operator's task except for the presence of actual *U*-boats. Therefore, even by the DoD's (2014) own standard, it would be generous to suggest the CDT is a passable simplification of real-life Cyber Defense Task demands.

The brevity of McIntire et al. (2013) 40-min-long trials also make the CDT's external validity unclear. In terms of laboratory vigilance investigations, 40 min is a typical period for performing a vigilance task (See et al., 1995; Helton et al., 1999; Warm et al., 2008, 2009; See, 2014). However, Chappelle et al. (2013) reported that active-duty cyber-defenders work for 51 h per week, or 10.5 h per day, with extremely limited rest breaks. Thus, the demands associated with a 40-min vigilance task are not analogous to a 10.5 h work day that Chappelle et al. (2013) observed to induce clinically significant levels of stress and burnout (O'Connell, 2012; Mancuso et al., 2015). By comparison to the rest of their day, the 40-min CDT could possibly have been a welcome respite for McIntire et al.'s (2013) the active service participants. It is hence unclear how externally valid the ocular changes that McIntire et al. (2013) associated with vigilance performance are, and how well these might extend across the standard 8–10-h shifts served by real-world cyber defenders.

The external validity of McIntire et al.'s (2013) study further suffered from insufficient control of confounding blue light exposure. A considerable proportion of the light emitted by many modern computer monitors is in the form of high-frequency blue light, and it is possible that the United States Air Force outfits their cyber defenders with these common tools (Lockley et al., 2006; Hatori et al., 2017). Blue light suppresses melatonin and actively increases the capacity to sustain attention on vigilance tasks in a dose-dependent fashion (Lockley et al., 2006; Holzman, 2010). Since this effect is dose-dependent, the longer cyber defenders are exposed to the blue light of their computer monitors, the greater vigilance performance could be expected to improve (Lockley et al., 2006). In a real-world cyber defense setting, analysts are exposed to 1,200 times the blue light exposure than the participants in McIntire et al. (2013). The vigilance performance enhancement provided by so much more blue light exposure may have rendered measuring the phenomenon far more than McIntire et al. (2013) suggested. Thus, the results reported by McIntire et al. (2013) may not generalize beyond the laboratory to the real-world (Reinerman-Jones et al., 2010; Hancock, 2013).

These largely technological critiques of the CDT's validity were overshadowed by the fact that McIntire et al.'s task was not validated according to Parasuraman's (1979, 1985) parameters of valid vigilance tasks. The first component of the CDT required that participants retain and recall three “suspicious” IP addresses from memory as they attempt each critical signal discrimination. This set of textual critical signals increased their participants' cognitive load while performing the CDT. However, because each critical CDT signal was considered in isolation, there was a gradual decline in cognitive load as time on the task increases. This is not the case in real world network defense. Operational analysts consider the alerts presented over their SEIM relative to one another within the wider virtual threat landscape (Heeger, 1997, 2007; Alserhani et al., 2010; Bridges, 2011; Majeed et al., 2019). For example, if a SEIM becomes flooded with benign alerts in a brief window of time, this can represent the beginning of a prevalence denial attack, as such, analysts must consider each benign alert in the context of all others presented by their system (Sawyer et al., 2016; Vieane et al., 2016). Cognitive load hence does not decline with time on task in operational network defense, whereas it does so in McIntire et al.'s (2013) CDT. It cannot therefore be claimed that vigilance decrement underlies the performance deficits observed by McIntire et al. (2013) on the CDT with any validity.

The frequency that alerts are presented to analysts by a SEIM is known as the event, or incident, rate (Simmons et al., 2013). The SEIM event rate communicates important information surrounding threatening elements distributed through the virtual threat landscape to analysts. For example, consider the rate that SEIM alerts occur at 2 am on Christmas Day against that observed at 11 am on a regular weekday. SEIM alerts are generally more frequent during the working week than during the holiday season (Pompon et al., 2018; Rodriguez and Okamura, 2019). Therefore, if the event rate at 2 am on Christmas Day even closely approximates that which is usually seen at 11 am on a weekday, this will influence how an analyst contextualizes and subsequently actions each SEIM alert. Even if every Christmas day SEIM alert is benign, the atypical event rate would influence the level of imminent risk perceived by an analyst in the virtual threat landscape (Vieane et al., 2016).

Event rate in real world network defense hence guides the way network defense analysts contextualize and then action SEIM alerts. This element of network defense was not captured by the CDT because McIntire et al. (2013) set the event rate to be a controlled variable. In an operational setting, analysts would also consider how quickly each “suspicious” IP address was presented in forming their threat level appraisal (Simmons et al., 2013). This further decreases the CDT's validity as a cyber vigilance task, as a fixed event rate may have impacted analysts' cognitive engagement with each potentially critical signal. That is, McIntire et al.'s (2013) participants needed to recruit fewer executive resources at a slower rate than their operational peers. It is therefore unclear if the performance deficits observed by McIntire et al. (2013) on the CDT resembled those observed during operational network defense.

Two types of critical signal were presented in the CDT, each via a different modality. The first type of critical signal was textual, in the form of three “suspicious” IP addresses that participants had to remember (McIntire et al., 2013). The second type of critical signal presented in the CDT was graphical and required no memory activation (McIntire et al., 2013). Although McIntire et al. (2013) had the requisite data to compare vigilance performance between the two critical signal modalities they did not report this comparison. Had vigilance performance varied between the graphical and textual critical signals, an argument could be made that this would demonstrate CDT performance sensitivity to signal salience. However, this would have been a tenuous argument at best, as the two signals were presented in vastly different ways. The CDT's textual critical signals were presented in a simultaneous fashion, which used participants' memory resources every time a discrimination was made. Simultaneous vigilance tasks require minimal executive resource activation because critical signal discriminations are based on sequential comparative judgements (Gartenberg et al., 2015, 2018). By comparison, the CDT's graphical critical signals were presented successively. Successive vigilance tasks are associated with a degree of cognitive workload above that of simultaneous tasks because operators must retain and recall critical signal information from memory before a discrimination can be made (Gartenberg et al., 2015, 2018). The primary deficiency of the CDT was fundamentally due to not being validated according to Parasuraman's (1979, 1985) vigilance task validity parameters. Similar deficiencies have also been found in Mancuso et al.'s (2015) Cyber Defense Task.

#### Mancuso et al.'s Cyber Defense Task (MCDT)

The MCDT presented network traffic logs in a waterfall display which their participants needed to read and action. Traffic logs contained four pieces of information, including two possible methods used to transmit data across the network, as well as the size, source, and destination of the transmission. A “signature” referred to a specific configuration of these four traffic log details that suggests malicious network activity. Mancuso et al.'s (2015) participants first needed to commit the details of a signature associated with a fictitious hacker to memory. They then had to identify any traffic log presented to them that matched at least three out of four items of the hacker's signature. The number of items within each log that matched the hacker's signature defined the color by which it was presented in the MCDT (Figure 1 in Mancuso et al., 2015). Mancuso et al. (2015) justified color coding each target to better resemble the systems used by the United States Air Force (Figure 1 in Mancuso et al., 2015). Logs that matched 0, 1, 2, 3, or all four elements of the hacker's signature were respectively colored, green, blue, violet, purple, and red in the MCDT. Of these, only purple and red logs were critical targets that the participant had to action.

#### Validity concerns with the MCDT

The MCDT was designed similarly to McIntire et al.'s (2013) CDT. For instance, the task maintained a fixed critical signal probability of 20%. However, fixed task demands such as this are difficult to generalize to real world operations (Helton et al., 2004). Primarily, this is because vigilance is sensitive to task demands, and in cyber defense, these fluctuate between great extremes (Helton et al., 2004; Chappelle et al., 2013).

Another questionable feature of the MCDT's validity is that the visual field of view is confined to a single computer monitor. In real world cyber security contexts, SEIMs require multiple monitors to portray the network's security status. Multiple monitors are pragmatically necessary due to the volume, diversity, and specificity of virtual threat data that analysts are required to handle (D'Amico et al., 2005). Hence, Mancuso et al.'s (2015) limited field of view restricted the range of cyber threat stimuli that could be sampled from real world operations for use in their cyber vigilance task. This detracted from the MCDT's external validity as a cyber vigilance task.

In addition, the color coding system that Mancuso et al. (2015) incorporated into the MCDT obscured the cognitive load participants experienced when discriminating between critical and non-critical traffic logs. For example, the volume and type of information required to discriminate critical MCDT traffic logs, both with and without color coding, is compared in Figure 1 in Mancuso et al. (2015).

Under the color coded system, participants needed to remember only two graphical elements of information, namely that the color of critical logs was indicated by red or purple (Table 1 and Figure 1 in Mancuso et al., 2015). This is in contrast with a colorless MCDT, where critical signals could only be identified when the participant remembered four elements of salient threat information in the hacker's signature. Because Mancuso et al.'s (2015) participants had two ways of interpreting the MCDT's signals, this made the cognitive load associated with the task unclear. There could be no way of knowing if Mancuso et al.'s (2015) participants analyzed each traffic log based on its color alone, or if they analyzed all four threat salient elements of information. Color coding the MCDT's signals therefore detracted from its external validity. That is, rather than bolstering the MCDT's external validity, Mancuso et al.'s (2015) color coding system instead served to confound the cognitive load associated with the task.

**Table 1:** Comparison of the MCDT with and without color coded signals.

**MCDT**	**Comparisons required to reach a decision**	**Critical signal decision rule**	**Critical signal working memory load**
Without color coding	Does the hacker's transmission method match the traffic log? Does the hacker's transmission size match the traffic log? Does the hacker's transmission source match the traffic log? Does the hacker's transmission destination match the traffic log?	If three out of four traffic log elements match the hacker's signature, then indicate the presence of a critical signal.	The participant needed to keep track of between 3 and 4 traffic log elements that might match the hacker's signature.
With color coding	Only red and purple colored traffic logs are critical. White, green, and blue traffic logs can be ignored.	If a traffic log is color coded as red or purple, then indicate the presence of a critical signal.	The participant only needed to remember two colors, red and purple

#### Sawyer et al.'s MCDT-II

Sawyer et al. (2016) used a modified form of the MCDT to investigate the impact of event rate and signal salience on cyber vigilance performance. For the purposes of discussion Sawyer et al.'s (2016) modified MCDT will be referred to as the MCDT-II. The MCDT-II presented network traffic logs to participants in a colorless waterfall display. In the original MCDT, these traffic logs detailed four threat salient pieces of information, namely, transmission method, size, source, and destination. Sawyer et al. (2016) adapted these traffic logs in the MCDT-II to include the source IP address, the source port, the destination IP address, and the destination port of each transmission (Figure 1 in Sawyer et al., 2016). Each network traffic log in the MCDT-II contained the IP address and communication port numbers for both the source and destination of a data transmission across a hypothetical network. Two new traffic logs appeared periodically at the top of the MCDT-II's display. The critical signal that participants needed be vigilant of was any instance in which a top row IP address and port number-pairs matched an existing traffic log already present on the display (see Figure 1 in Sawyer et al., 2016).

Unlike McIntire et al. (2013) and Mancuso et al. (2015), Sawyer et al. (2016) attempted to validate their cyber vigilance task according to two of Parasuraman's (1979, 1985) parameters, namely, event rate and signal salience. Sawyer et al. (2016) formed four experimental conditions based on two levels of event rate and signal salience, respectively (Table 2). Sawyer et al. (2016) reported reductions in vigilance performance when critical MCDT-II signals were low in signal salience, slowly presented, or both. Sawyer et al. (2016) observed a gradual decline in the mean percentage of correctly identified MCDT-II signals. Moreover, in accordance with Parasuraman (1979, 1985), Sawyer et al. (2016) found that these reductions in performance were mediated by the signal salience and event rate of the MCDT-II.

**Table 2:** Levels of event rate and signal salience examined by Sawyer et al. (2016).

**Signal salience**	**Event rate**	**Condition**
Low (5% chance).	Slow (eight events per minute).	Low.Slow.
	Fast (16 events per minute).	Low.Fast.
High (20% chance).	Slow (eight events per minute).	High.Slow.
	Fast (16 events per minute).	High.Fast.

**Figure 1:**
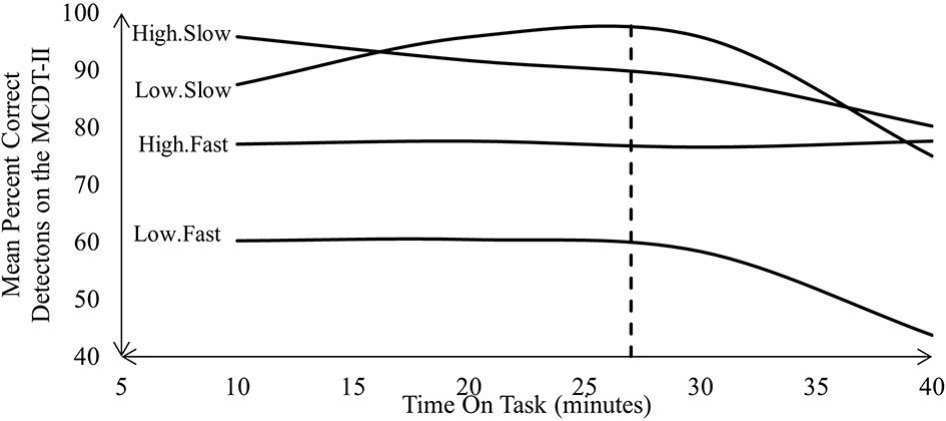
MCDT-II performance Sawyer et al. (2016) reported.

With the possible exception of the High.Fast condition, Sawyer observed changes in vigilance performance that align with vigilance decrement (Figure 1). Each condition Sawyer et al. (2016) tested was composed of variations in event rate and signal salience. Sawyer et al. (2016) observed that event rate had a greater influence over vigilance performance at baseline than signal salience. For example, vigilance performance under both slow conditions was higher than in the fast conditions after 10 min. However, signal salience appeared to have the greater influence by the end of the trial. For example vigilance performance in both slow and fast high signal salience condition outperformed what Sawyer et al. (2016) observed in the low signal salience condition. Sawyer et al. (2016) also reported variations in signal salience and event rate influenced trajectory of vigilance performance across all four conditions. For example, after ~30 min, Sawyer et al. (2016) reported sharp declines in the trajectory of vigilance performance observed under both low signal salience conditions (Figure 1). In contrast, Sawyer et al. (2016) reported more linear declines in vigilance performance under the high signal salienc econditions. However, this linear decline varied drastically between the High.Slow and High.Fast conditions. For example, vigilance performance under the High.Fast condition only changed by 0.52% from baseline. In contrast, vigilance performance under the High.Slow condition dropped by 15.62%, which more closely approximates the average decline across all conditions, which came to ~14.85%.

Differing compositions of signal salience and event rate also resulted in clear level differences in vigilance performance. For example, vigilance performance in the Low.Fast condition was the lowest acros the entire duration of the task, and also had the lowest final final value. By the end of the task, the level of the High.Slow, Low.Slow and High.Fast vigilance performance curves all appear approximately similar at around 77.5%. The only exception to this was the value of the Low. Fast condition, which ended at almost half of all other conditions, at 43.75%. Sawyer et al. (2016) therefore demonstrated that variations in event rate and signal salience influenced the way vigilance decrement presented throughout the entire MCDT-II. Sensitivity to signal salience and event rate are just two of Parasuraman's (1979, 1985) three parameters that characterize a valid vigilance task. Sensitivity to cognitive load was Parasuraman's (1979, 1985) third parameter of a valid vigilance, which was a controlled variable in Sawyer et al. (2016). The MCDT-II was therefore only partially validated as a cyber vigilance task.

## Challenges of developing cyber vigilance tasks

### Access and confidentiality

Like many security sub domains, network defense analysts and their workplaces can be difficult to access for the purposes of research (Paul, 2014; Gutzwiller et al., 2015). It can therefore be difficult to obtain details about Cyber Security Operations Centers' operational procedures or SEIM software console, as these are extremely sensitive corporate information that many enterprises would be hesitant about sharing with outsiders (Paul, 2014). This information is, however, crucial to the development of a cyber vigilance task. Access and confidentiality can therefore hinder the process of designing a vigilance task that accurately parallels the operational cognitive demands of network defense (Paul, 2014). In contrast, Mackworth (1948, 1950) was able to rely on support from the Royal Air Force to create his formative clock vigilance task. For example, the Royal Air Force granted Mackworth direct access to their radar equipment and operators, at a time in history where this critical strategic information would have been closely guarded in Europe after World War Two.

### Task complexity

The sheer complexity of cyber security may also explain why there are so few vigilance tasks for network defense in the literature. That is, simulating the complex demands of operational network defense is central to the development of a generalizable cyber vigilance task (Reinerman-Jones et al., 2010; Hancock, 2013). This is because the behavioral presentation of vigilance decrement functions according to the domain specific demands of the task being performed (Donald, 2008; Reinerman-Jones et al., 2010; Hancock, 2013). That is, if the demands of an operational vigilance task are not accurately captured by its laboratory analog, then the behavioral presentation of any performance decrement that occurs may not generalize to the operational setting (Donald, 2008; Reinerman-Jones et al., 2010; Hancock, 2013). The predictive validity of laboratory-based vigilance research hence hinges on the degree to which task demands match what is observed operationally (Donald, 2008; Reinerman-Jones et al., 2010; Hancock, 2013; Gutzwiller et al., 2015).

### Non-standard operating environments

The absence of a validated cyber vigilance task in the literature may also be explained by the fact that network defense analysts are known to customize their work terminals. SEIMs integrate cyber threat intelligence, derived from inbound and outbound network traffic, and present this to analysts, who then action appropriate defensive responses to virtual threats (Tresh and Kovalsky, 2018).

SEIMS are built according to the diverse cyber security needs of specific organizations, and are not engineered according to a common, standardized design. In contrast, Mackworth (1948, 1950) was able to derived the clock task from real world radar display that was characterized by a standardized design. However, SEIM's are not designed according to a standardized design, and as such, it was not possible to derive a modern cyber vigilance task from a given SEIM in industry in the same way Mackworth's (1948, 1950) clocks were based on real world radars (Work, 2020).

Further complicating the challenge of designing a modern cyber vigilance task, in addition to non-standard SEIM designs, is the fact that many analysts also customize their personal workstations, a practice that produces radical differences in task performance even within the same cyber security team (Hao et al., 2013). These customisations alter the cognitive load required to use a SEIM, which in turn can alter the behavioral presentation of vigilance decrement.

### Rapid obsolescence

Like many technology subfields, cyber security is evolving quickly (Gutzwiller et al., 2015). Moreover, the rate of evolution in cyber security is unlike the rate in any other domain in which vigilance decrement has been observed. Rapid evolution in the technological complexity of cyber security may also explain why the literature lacks a modern vigilance task for network defense. Cyber vigilance tasks can become obsolete experimental tools as quickly as the systems they have been designed to emulate (Gutzwiller et al., 2015). For example, although cars vary in the design and layout of their control surfaces, driving has remained a fundamentally unchanged task for decades. In turn, driver vigilance tasks have likewise remained fundamentally the same for decades (Milakis et al., 2015). Hence, unlike cyber security, the validity of driver vigilance tasks is unlikely to degrade over time, as the fundamental elements of the task are also unlikely to change significantly (Gutzwiller et al., 2015).

Cyber security's rapid evolution therefore limits the long-term validity of any vigilance task designed for the space. For example, the single computer monitor used to run McIntire et al.'s (2013) cyber vigilance task shows its age. In comparison to 2013, modern network defense is too complex a task to complete on a single computer monitor, which forces analysts to divide their attention across multiple screens of information (D'Amico et al., 2005; Axon et al., 2018). This difference in required screen real estate reflects an evolution in the volume of information that human operators are required to handle in the defense of a network. This in turn reflects growth in the level of cognitive load that analysts must sustain as they hunt for threats distributed across the virtual threat landscape. McIntire et al.'s (2013) single-screen cyber vigilance task therefore inaccurately simulated the demands associated with modern network defense. Furthermore, this suggests that the validity of cyber vigilance tasks may be sensitive to the rapid rate at which the technological tools develop in this space.

Tasks that require routine updates to remain valid are not uncommon in the psychological space. For example, the Wechsler Adult Intelligence Scale is an established psychometric instrument that requires routine updates to minimize reduced validity (Wechsler, 2002). Cyber vigilance tasks might likewise require periodic updates to maintain valid simulators of network defense. Hence McIntire et al.'s (2013) CDT may have reasonably approximated the demands of network security at the time it was published. However, by the standards of modern network defense, McIntire et al.'s (2013) task is outdated. Had the CDT been updated periodically to keep up with developments in network security, this would have preserved some degree of its validity as a vigilance task.

Table 3 summarizes the various challenges McIntire et al. (2013), Mancuso et al. (2015), and Sawyer et al. (2016) encountered in creating a cyber vigilance task. These are challenges future researchers will need to navigate if the gap in the literature left by a modern, validated cyber vigilance task is to ever be addressed.

**Table 3:** Cyber vigilance task creation challenges.

**Challenge**	**Challenge mitigation**
Access and confidentiality	Gaining access to cyber security organizations and personnel can limit the process of designing and subsequently testing cyber vigilance tasks. However, McIntire et al. (2013), Mancuso et al. (2015), and Sawyer et al. (2016) demonstrated this challenge can be navigated by performing research with cyber industry partners.
Task complexity	The CDT, MCDT, and MCDT-II that McIntire et al. (2013), Mancuso et al. (2015), and Sawyer et al. (2016) were all oversimplified emulations of network defense consoles, which did not accurately simulate the cognitive demands associated with real world cyber security.
Non-standard operating environments	It is not possible to base the design of any cyber vigilance task on an operational SEIM, because no single console is standardized across industry.
Rapid obsolescence	The pace of technological evolution in cyber security means that the validity of cyber vigilance tasks has a shelf life. As network defense technologies grow increasingly complex, this require consistently updating and revalidating cyber vigilance tasks.

## Conclusion

In closing, vigilance decrement is a cyber-cognitive vulnerability which must be better understood to manage it as a human factor security risk. However, advancing our understanding of vigilance decrement in the network defense space necessitates developing experimental testbeds that accommodate access and confidentiality, task complexity, non-standard operating environments, and rapid obsolescence. Moving forward, improving the interaction between SEIM consoles and human network defense analysts, necessitates developing an updated cyber vigilance task that is also valid according to Parasuraman's (1979, 1985) parameters.

## Author contributions

All authors listed have made a substantial, direct, and intellectual contribution to the work and approved it for publication.

## References

[B1] Aidman E. Chadunow C. Johnson K. Reece J. (2015). Real-time driver drowsiness feedback improves driver alertness and self-reported driving performance. Accid. Anal. Prev. 81, 8–13. 10.1016/j.aap.2015.03.041 25932964

[B2] Albayati M. Issac B. (2015). Analysis of intelligent classifiers and enhancing the detection accuracy for intrusion detection system. Int. J. Comput. Intell. Syst. 8, 841–853. 10.1080/18756891.2015.108470535746198

[B3] Aleem A. Ryan Sprott C. (2013). Let me in the cloud: Analysis of the benefit and risk assessment of cloud platform. J. Fin. Crime 20, 6–24. 10.1108/13590791311287337

[B4] Alhawari S. Karadsheh L. Talet A. N. Mansour E. (2012). Knowledge-based risk management framework for information technology project. Int. J. Informat. Manag. 32, 50–65. 10.1016/j.ijinfomgt.2011.07.00219745243

[B5] Alserhani F. Akhlaq M. Awan I. U. Cullen A. J. Mirchandani P. (2010). “MARS: Multi-stage attack recognition system 2010,” in 24th IEEE International Conference on Advanced Information Networking and Applications (Perth). 10.1109/AINA.2010.57

[B6] Al-Shargie F. Tariq U. Mir H. Alawar H. Babiloni F. Al-Nashash H. (2019). Vigilance decrement and enhancement techniques: A review. Brain Sci. 9, 178. 10.3390/brainsci9080178 31357524 PMC6721323

[B7] Axon L. Alahmadi B. Nurse J. Goldsmith M. Creese S. (2018). “Sonification in security operations centres: What do security practitioners think?,” The Network and Distributed System Security (NDSS) Symposium 2018. San Diego, CA. 10.14722/usec.2018.23024

[B8] Baker C. (1959). Attention to visual displays during a vigilance task: II. Maintaining the level of vigilance. Br. J. Psychol. 50, 30–36. 10.1111/j.2044-8295.1959.tb00678.x 13628967

[B9] Barford P. Dacier M. Dietterich T. G. Fredrikson M. Giffin J. Jajodia S.. (2010). “Cyber SA: Situational awareness for cyber defense,” in Cyber Situational Awareness (Berlin: Springer), 3–13. 10.1007/978-1-4419-0140-8_1

[B10] Ben-Asher N. Gonzalez C. (2015). Effects of cyber security knowledge on attack detection. Comput. Hum. Behav. 48, 51–61. 10.1016/j.chb.2015.01.039

[B11] Bodala I. P. Li J. Thakor N. V. Al-Nashash H. (2016). EEG and eye tracking demonstrate vigilance enhancement with challenge integration. Front. Hum. Neurosci. 10, 273. 10.3389/fnhum.2016.00273 27375464 PMC4894919

[B12] Bridges N. R. (2011). Predicting Vigilance Performance Under Transcranial Direct Current Stimulation (Publication Number 1047). (Masters Thesis), Wright State University, Dayton, OH. Available online at: https://corescholar.libraries.wright.edu/etd_all/1047/ (accessed March 6, 2020).

[B13] Cabrall C. Happee R. De Winter J. (2016). From Mackworth's clock to the open road: A literature review on driver vigilance task operationalization. Transport. Res. F 40, 169–189. 10.1016/j.trf.2016.04.001

[B14] Cavelty M. D. (2014). Breaking the cyber-security dilemma: Aligning security needs and removing vulnerabilities. Sci. Eng. Ethics 20, 701–715. 10.1007/s11948-014-9551-y 24781874

[B15] Chappelle W. McDonald K. Christensen J. Prince L. Goodman T. Thompson W. (2013). Sources of Occupational Stress and Prevalence of Burnout and Clinical Distress Among US Air Force Cyber Warfare Operators [Final Technical Report] (88ABW-2013-2089). Available online at: https://apps.dtic.mil/dtic/tr/fulltext/u2/a584653.pdf (accessed March 6, 2020).

[B16] Cunningham S. G. Freeman F. (1994). The Electrocortical Correlates of Fluctuating States of Attention During Vigilance Tasks [Contractor Report (CR)](19950008450). (NASA Contractor Report – NASA-CR-197051., NASA Contractor Report – NASA CR-197051, Issue. Available online at: https://ntrs.nasa.gov/api/citations/19950008450/downloads/19950008450.pdf (accessed March 7, 2020).

[B17] Daly T. Murphy J. Anglin K. Szalma J. Acree M. Landsberg C.. (2017). “Moving vigilance out of the laboratory: Dynamic scenarios for UAS operator vigilance training,” in Augmented Cognition. Enhancing Cognition and Behavior in Complex Human Environments (Berlin: Springer International Publishing), 20–35. 10.1007/978-3-319-58625-0_2

[B18] D'Amico A. Whitley K. Tesone D. O'Brien B. Roth E. (2005). Achieving cyber defense situational awareness: A cognitive task analysis of information assurance analysts. Proc. Hum. Fact. Ergon. Soc. Ann. Meet. 49, 229–233. 10.1177/154193120504900304

[B19] Dember W. N. Galinsky T. L. Warm J. S. (1992). The role of choice in vigilance performance. Bullet. Psychon. Soc. 30, 201–204. 10.3758/BF03330441

[B20] DoD (2014). Mission Analysis for Cyber Operations of Department of Defense (E-0CD45F6). Available online at: https://info.publicintelligence.net/DoD-CyberMissionAnalysis.pdf (accessed April 4, 2020).

[B21] Donald F. M. (2008). The classification of vigilance tasks in the real world. Ergonomics 51, 1643–1655. 10.1080/00140130802327219 18941972

[B22] D'Orazio T. Leo M. Guaragnella C. Distante A. (2007). A visual approach for driver inattention detection. Patt. Recogn. 40, 2341–2355. 10.1016/j.patcog.2007.01.018

[B23] Endsley M. Kiris E. (1995). The out-of-the-loop performance problem and level of control in automation. Hum. Fact. 37, 32–64. 10.1518/00187209577904954329683404

[B24] Epling S. L. Russell P. N. Helton W. S. (2016). A new semantic vigilance task: Vigilance decrement, workload, and sensitivity to dual-task costs. Exp. Brain Res. 234, 133–139. 10.1007/s00221-015-4444-0 26403293

[B25] Erola A. Agrafiotis I. Happa J. Goldsmith M. Creese S. Legg P. A. (2017). “RicherPicture: Semi-automated cyber defence using context-aware data analytics,” in The 2017 International Conference On Cyber Situational Awareness, Data Analytics And Assessment (Cyber SA) (London) 10.1109/CyberSA.2017.8073399

[B26] Fathi N. Mehraban A. H. Akbarfahimi M. Mirzaie H. (2017). Validity and reliability of the test of everyday attention for children (teach) in Iranian 8-11 year old normal students. Iran. J. Psychiatr. Behav. Sci. 11, 1–7. 10.5812/ijpbs.2854

[B27] Finomore V. Matthews G. Shaw T. Warm J. (2009). Predicting vigilance: A fresh look at an old problem. Ergonomics 52, 791–808. 10.1080/00140130802641627 19562590

[B28] Franke U. Brynielsson J. (2014). Cyber situational awareness – A systematic review of the literature. Comput. Secur. 46, 18–31. 10.1016/j.cose.2014.06.008

[B29] Gartenberg D. Gunzelmann G. Hassanzadeh-Behbaha S. Trafton J. G. (2018). Examining the role of task requirements in the magnitude of the vigilance decrement. Front. Psychol. 9, 1504. 10.3389/fpsyg.2018.01504 30177902 PMC6109784

[B30] Gartenberg D. Gunzelmann G. Veksler B. Z. Trafton J. G. (2015). “Improving vigilance analysis methodology: questioning the successive versus simultaneous distinction,” in Proceedings of the Human Factors and Ergonomics Society Annual Meeting (Los Angeles, CA) 10.1177/1541931215591059

[B31] Gaw T. J. (2014). ARL-VIDS Visualization Techniques: 3D Information Visualization of Network Security Events (Publication Number 882577849). (Masters Thesis), Ball State University, Muncie, IN. Available online at: http://liblink.bsu.edu/catkey/1745749 (accessed April 1, 2020).

[B32] Gopalakrishnan S. (2012). A public health perspective of road traffic accidents. J. Fam. Med. Primary Care 1, 144–150. 10.4103/2249-4863.104987 24479025 PMC3893966

[B33] Gordon L. A. Loeb M. P. Zhou L. (2011). The impact of information security breaches: Has there been a downward shift in costs? J. Comput. Secur. 19, 33–56. 10.3233/JCS-2009-0398

[B34] Goutam R. K. (2015). Importance of cyber security. Int. J. Comput. Appl. 111, 1250. 10.5120/19550-1250

[B35] Gutzwiller R. S. Fugate S. Sawyer B. D. Hancock P. (2015). “The human factors of cyber network defense,” in Proceedings of the Human Factors and Ergonomics Society Annual Meeting. (Los Angeles, CA). 10.1177/1541931215591067

[B36] Gutzwiller R. S. Hunt S. M. Lange D. S. (2016). “A task analysis toward characterizing cyber-cognitive situation awareness (CCSA) in cyber defense analysts,” in The 2016 IEEE International Multi-Disciplinary Conference on Cognitive Methods in Situation Awareness and Decision Support (CogSIMA). (San Diego, CA). 10.1109/COGSIMA.2016.7497780

[B37] Hancock P. A. (2013). In search of vigilance: The problem of iatrogenically created psychological phenomena. Am. Psycholog. 68, 97–109. 10.1037/a0030214 23088439

[B38] Hancock P. A. Hart S. (2002). Defeating terrorism: What can human factors/ergonomics offer? Ergon. Design 10, 6–16. 10.1177/106480460201000103

[B39] Hao L. Healey C. G. Hutchinson S. E. (2013). Flexible web visualization for alert-based network security analytics. Assoc. Comput. Machinery. 10.1145/2517957.2517962

[B40] Hatori M. Gronfier C. Van Gelder R. N. Bernstein P. S. Carreras J. Panda S.. (2017). Global rise of potential health hazards caused by blue light-induced circadian disruption in modern aging societies. NPJ Aging Mechanisms Dis. 3, 1–3. 10.1038/s41514-017-0010-2 28649427 PMC5473809

[B41] Heeger D. (1997). Signal Detection Theory. New York University. Available online at: https://www.cns.nyu.edu/~david/handouts/sdt/sdt.html (accessed May 31, 2020).

[B42] Heeger D. (2007). Signal Detection Theory. New York University. Available online at: https://www.cns.nyu.edu/david/handouts/sdt/sdt.html (accessed May 31, 2020).

[B43] Helton W. S. Dember W. N. Warm J. S. Matthews G. (1999). Optimism, pessimism, and false failure feedback: Effects on vigilance performance. Curr. Psychol. 18, 311–325. 10.1007/s12144-999-1006-2

[B44] Helton W. S. Shaw T. H. Warm J. S. Dember G. M. W. N. Hancock P. A. (2004). “Demand transitions in vigilance: Effects on performance efficiency and stress,” in Human Performance, Situation Awareness, and Automation: Current Research and Trends HPSAA II, Volumes I and II, eds V. M. Mouloua and P. A. Hancock (Mahwah, NJ: Lawrence Erlbaum Associates, Inc., Publishers), 258–263.

[B45] Hicks J. M. (2015). A Theater-Level Perspective on Cyber (0704-0188). N. D. U. Press. Available online at: https://apps.dtic.mil/dtic/tr/fulltext/u2/a618537.pdf (accessed April 3, 2020).

[B46] Holzman D. C. (2010). What's in a color? The unique human health effects of blue light. Environ. Health Perspect. 118, 22–27. 10.1289/ehp.118-a22 20061218 PMC2831986

[B47] Jo J. Lee S. J. Park K. R. Kim I.-J. Kim J. (2014). Detecting driver drowsiness using feature-level fusion and user-specific classification. Expert Syst. Appl. 41, 1139–1152. 10.1016/j.eswa.2013.07.108

[B48] Jolley J. D. (2012). Article 2 and Cyber Warfare: How Do Old Rules Control the Brave New World? Available at SSRN 2128301. 2. World Wide Organisation; Institution of Engineering and Technology. 1–16. 10.5539/ilr.v2n1p1

[B49] Joly A. Zheng R. Kaizuka T. Nakano K. (2017). Effect of drowsiness on mechanical arm admittance and driving performances. Inst. Eng. Technol. Intell. Transport Syst. 12, 220–226. 10.1049/iet-its.2016.0249

[B50] Komlodi A. Goodall J. R. Lutters W. G. (2004). “An information visualization framework for intrusion detection,” in Association for Computing Machinery 2004 Conference on Human Factors in Computing Systems. (Vienna). 10.1145/985921.1062935

[B51] Lockley S. W. Evans E. E. Scheer F. A. Brainard G. C. Czeisler C. A. Aeschbach D. (2006). Short-wavelength sensitivity for the direct effects of light on alertness, vigilance, and the waking electroencephalogram in humans. Sleep 29, 161–168. 10.1093/sleep/29.2.161 16494083

[B52] Mackworth J. F. (1968). Vigilance, arousal, and habituation. Psychol. Rev. 4, 308–322. 10.1037/h0025896 4875885

[B53] Mackworth N. H. (1948). The breakdown of vigilance during prolonged visual search. Quart. J. Exp. Psychol. 1, 6–21. 10.1080/17470214808416738

[B54] Mackworth N. H. (1950). Researches on the measurement of human performance. J. Royal Stat. Soc. Ser. A. 113, 588–589. 10.2307/2980885

[B55] Majeed A. ur Rasool R. Ahmad F. Alam M. Javaid N. (2019). Near-miss situation based visual analysis of SIEM rules for real time network security monitoring. J. Ambient Intell. Human. Comput. 10, 1509–1526. 10.1007/s12652-018-0936-7

[B56] Mancuso V. F. Christensen J. C. Cowley J. Finomore V. Gonzalez C. Knott B. (2014). “Human factors in cyber warfare II: Emerging perspectives,” in Proceedings of the Human Factors and Ergonomics Society Annual Meeting. (Chicago, IL). 10.1177/1541931214581085

[B57] Mancuso V. F. Greenlee E. T. Funke G. Dukes A. Menke L. Brown R.. (2015). Augmenting cyber defender performance and workload through sonified displays. Proc. Manufact. 3, 5214–5221. 10.1016/j.promfg.2015.07.589

[B58] Maybury M. T. (2012). “Air force cyber vision 2025,” in 5th International Symposium on Resilient Control Systems. Salt Lake City, UT.

[B59] McIntire L. McKinley R. A. McIntire J. Goodyear C. Nelson J. (2013). Eye metrics: An alternative vigilance detector for military operators. Milit. Psychol. 25, 502–513. 10.1037/mil0000011

[B60] Milakis D. Van Arem B. Van Wee B. (2015). The Ripple Effect of Automated Driving BIVEC-GIBET Transport Research Day, May 28–29. 2015, Eindhoven, The Netherlands. Available online at: http://resolver.tudelft.nl/uuid:e6ecff79-4334-4baa-a60b-3ed897587157 (accessed April 3, 2020).

[B61] Nanay B. (2018). Perception is not all-purpose. Synthese 1, 1–12. 10.1007/s11229-018-01937-5 34720226 PMC8550056

[B62] Newcomb E. A. Hammell R. J. (2016). “A fuzzy logic utility framework (FLUF) to support information assurance,” in Software Engineering Research, Management and Applications, ed R. Lee (Berlin: Springer), 33–48. 10.1007/978-3-319-33903-0_3

[B63] O'Connell M. E. (2012). Cyber security without cyber war. J. Conflict Secur. Law 17, 187–209. 10.1093/jcsl/krs017

[B64] Ormrod D. (2014). “The coordination of cyber and kinetic deception for operational effect: Attacking the C4ISR interface,” in The 2014 IEEE Military Communications Conference. Baltimore, MD.

[B65] Parasuraman R. (1979). Memory load and event rate control sensitivity decrements in sustained attention. Science 205, 924–927. 10.1126/science.472714 472714

[B66] Parasuraman R. (1985). “Sustained attention: A multifactorial approach,” in Attention and Performance XI, Vol. 1482, ed M. I. Posner and M. S. Oscar (Mahwah, NJ: Lawrence Erlbaum Associates, Inc., Publishers), 493–511.

[B67] Parasuraman R. Mouloua M. (1987). Interaction of signal discriminability and task type in vigilance decrement. Percept. Psychophys. 41, 17–22. 10.3758/BF03208208 3822739

[B68] Pattyn N. Neyt X. Henderickx D. Soetens E. (2008). Psychophysiological investigation of vigilance decrement: Boredom or cognitive fatigue? Physiol. Behav. 93, 369–378. 10.1016/j.physbeh.2007.09.016 17999934

[B69] Paul C. L. (2014). “Human-centered study of a network operations center: Experience report and lessons learned,” in Proceedings of the 2014 ACM Workshop on Security Information Workers (New York, NY). 10.1145/2663887.2663899

[B70] Pompon R. Walkowski D. Boddy S. Levin M. (2018). 2018 Phishing and Fraud Report - Attacks Peak During The Holidays (Phishing and Fraud Report, Issue. F. Labs). Available online at: https://www.f5.com/labs/articles/threat-intelligence/2018-phishing-and-fraud-report–attacks-peak-during-the-holidays (accessed April 3, 2020).

[B71] Pradhapan P. Griffioen R. Clerx M. Mihajlović V. (2017). “Personalized characterization of sustained attention/vigilance in healthy children,” in Lecture Notes of the Institute for Computer Sciences, Social Informatics and Telecommunications Engineering, Vol. 181, eds K. Giokas, L. Bokor, and F. Hopfgartner (Cham: Springer International Publishing), 271–281. 10.1007/978-3-319-49655-9_35

[B72] Rajan A. V. Ravikumar R. Al Shaer M. (2017). “UAE cybercrime law and cybercrimes—An analysis,” in The 2017 International Conference on Cyber Security And Protection Of Digital Services (Cyber Security). 10.1109/CyberSecPODS.2017.8074858

[B73] Rajivan P. Cooke N. (2017). “Impact of team collaboration on cybersecurity situational awareness,” in Theory and Models for Cyber Situation Awareness, eds P. Liu, S. Jajodia, and C. Wang (Cham: Springer International Publishing), 203–226. 10.1007/978-3-319-61152-5_8

[B74] Rajivan P. Janssen M. A. Cooke N. J. (2013). “Agent-based model of a cyber security defense analyst team,” in Proceedings of the Human Factors and Ergonomics Society Annual Meeting (San Diego, CA), 10.1177/1541931213571069

[B75] Reinerman-Jones L. Matthews G. Mercado J. E. (2016). Detection tasks in nuclear power plant operation: Vigilance decrement and physiological workload monitoring. Saf. Sci. 88, 97–107. 10.1016/j.ssci.2016.05.002

[B76] Reinerman-Jones L. E. Matthews G. Langheim L. K. Warm J. S. (2010). Selection for vigilance assignments: A review and proposed new direction. Theoret. Iss. Ergon. Sci. 12, 273–296. 10.1080/14639221003622620

[B77] Rodriguez A. Okamura K. (2019). “Generating real time cyber situational awareness information through social media data mining,” in 2019 IEEE 43rd Annual Computer Software and Applications Conference (COMPSAC). (Milwaukee, WI). 10.1109/COMPSAC.2019.10256

[B78] Saltzman I. (2013). Cyber posturing and the offense-defense balance. Contemp. Secur. Pol. 34, 40–63. 10.1080/13523260.2013.771031

[B79] Satterfield K. Harwood A. E. Helton W. S. Shaw T. H. (2019). Does depleting self-control result in poorer vigilance performance? Hum. Fact. 61, 415–425. 10.1177/0018720818806151 30372632

[B80] Sawyer B. D. Finomore V. S. Funke G. J. Matthews G. Mancuso V. Funke M. (2016). Cyber Vigilance: The Human Factor (0704-0188). Available online at: https://apps.dtic.mil/sti/pdfs/AD1021913.pdf (accessed April 4, 2020).

[B81] Sawyer B. D. Hancock P. A. (2018). Hacking the human: The prevalence paradox in cybersecurity. Hum. Fact. 60, 597–609. 10.1177/0018720818780472 29986155

[B82] See J. E. (2014). Vigilance: A Review of the Literature and Applications to Sentry Duty (SAND2014-17929). United States: Office of Scientific and Technical Information (OSTI). 10.2172/1322275

[B83] See J. E. Howe S. R. Warm J. S. Dember W. N. (1995). Meta-analysis of the sensitivity decrement in vigilance. Psychol. Bullet. 117, 230–249. 10.1037/0033-2909.117.2.230

[B84] Sherwood M. S. Kane J. H. Weisend M. P. Parker J. G. (2016). Enhanced control of dorsolateral prefrontal cortex neurophysiology with real-time functional magnetic resonance imaging (rt-fMRI) neurofeedback training and working memory practice. Neuroimage 124, 214–223. 10.1016/j.neuroimage.2015.08.074 26348555

[B85] Simmons C. B. Shiva S. G. Bedi H. S. Shandilya V. (2013). “ADAPT: A game inspired attack-defense and performance metric taxonomy,” in IFIP International Information Security Conference (Memphis, MS). 10.1007/978-3-642-39218-4_26

[B86] Skopik F. Settanni G. Fiedler R. (2016). A problem shared is a problem halved: A survey on the dimensions of collective cyber defense through security information sharing. Comput. Secur. 60, 154–176. 10.1016/j.cose.2016.04.003

[B87] Smith M. (2016). “The Effect of Perceived Humanness in Non-Human Robot Agents on Social Facilitation in a Vigilance Task (Publication Number 10132069). (Doctoral dissertation), George Mason University, Fairfax, VA. Available online at: https://search.proquest.com/openview/49fba8a8ccd3001dd6465ccb7bddbd70/1?pq-origsite=gscholar&cbl=18750&diss=y (accessed April 5, 2020).

[B88] Sommer D. Golz M. (2010). “Evaluation of PERCLOS based current fatigue monitoring technologies,” *The 2010 Annual International Conference of the IEEE Engineering in Medicine and Biology* (Buenos Aires). 10.1109/IEMBS.2010.5625960 21095770

[B89] Sostek A. J. (1978). Effects of electrodermal lability and payoff instructions on vigilance performance. Psychophysiology 15, 561–568. 10.1111/j.1469-8986.1978.tb03110.x 715124

[B90] Spathoulas G. P. Katsikas S. K. (2010). Reducing false positives in intrusion detection systems. Comput. Secur. 29, 35–44. 10.1016/j.cose.2009.07.008

[B91] Spathoulas G. P. Katsikas S. K. (2013). Enhancing IDS performance through comprehensive alert post-processing. Comput. Secur. 37, 176–196. 10.1016/j.cose.2013.03.005

[B92] St John M. Risser M. R. Kobus D. A. (2006). “Toward a usable closed-loop attention management system: Predicting vigilance from minimal contact head, eye, and EEG measures,” in Proceedings of the 2nd Annual Augmented Cognition, San Franciso, CA. Available online at: http://citeseerx.ist.psu.edu/viewdoc/download?doi=10.1.1.135.1229&rep=rep1&type=pdf (accessed April 5, 2020).

[B93] Tan H. Zhang Y.-J. (2006). Detecting eye blink states by tracking iris and eyelids. Pat. Recogn. Lett. 27, 667–675. 10.1016/j.patrec.2005.10.005

[B94] Thiffault P. Bergeron J. (2003a). Fatigue and individual differences in monotonous simulated driving. Personal. Individ. Diff. 34, 159–176. 10.1016/S0191-8869(02)00119-8

[B95] Thiffault P. Bergeron J. (2003b). Monotony of road environment and driver fatigue: A simulator study. Accid. Anal. Prev. 35, 381–391. 10.1016/S0001-4575(02)00014-3 12643955

[B96] Thomason S. (2013). People–The weak link in security. Glob. J. Comput. Sci. Technol.

[B97] Tian H. T. Huang L. S. Zhou Z. Luo Y. L. (2004). “Arm up administrators: Automated vulnerability management,” in 7th International Symposium on Parallel Architectures, Algorithms and Networks, 2004. Proceedings (Hong Kong). 10.1109/ISPAN.2004.1300542

[B98] Tong M. Thomson C. (2015). “Developing a critical literature review for project management research,” in Designs, Methods and Practices for Research of Project Management. (London: Gower Publishing Limited; Routledge), 153–171).

[B99] Tresh K. Kovalsky M. (2018). Toward Automated Information Sharing California: Cybersecurity Integration Center's approach to improve on the traditional information sharing models. Cyber Defense Rev. 3, 23–32. Available online at: https://www.jstor.org/stable/26491220

[B100] Tyworth M. Giacobe N. A. Mancuso V. (2012). Cyber situation awareness as distributed socio-cognitive work. Cyber Sens. 2012, 919338. 10.1117/12.919338

[B101] Valdez P. (2019). Homeostatic and circadian regulation of cognitive performance. Biolog. Rhythm Res. 50, 85–93. 10.1080/09291016.2018.149127133162871

[B102] Vieane A. Funke G. Gutzwiller R. Mancuso V. Sawyer B. Wickens C. (2016). “Addressing human factors gaps in cyber defense,” in Proceedings of the Human Factors and Ergonomics Society Annual Meeting (Washington, DC). 10.1177/1541931213601176

[B103] Wall D. S. Williams M. L. (2013). Policing cybercrime: Networked and social media technologies and the challenges for policing. Policing Soc. 23, 409–412. 10.1080/10439463.2013.780222

[B104] Warm J. S. Dember W. (1998). “Tests of vigilance taxonomy,” in Viewing Psychology as a Whole: The Integrative Science of William N. Dember, eds R. R. Hoffman and M. F. Sherrick (Washington, DC: American Psychological Association). 10.1037/10290-004

[B105] Warm J. S. Matthews G. Finomore V. S. (2018). “Vigilance, workload, and stress,” in Performance Under Stress, eds P. A. Hancock and J. L. Szalma (Boca Raton, FL: CRC Press), 131–158.

[B106] Warm J. S. Matthews G. Parasuraman R. (2009). Cerebral hemodynamics and vigilance performance. Milit. Psychol. 21, 75–100. 10.1080/08995600802554706

[B107] Warm J. S. Parasuraman R. Matthews G. (2008). Vigilance requires hard mental work and is stressful. Hum. Fact. 50, 433–441. 10.1518/001872008X312152 18689050

[B108] Wechsler D. (2002). Technical Manual (Updated) for the Wechsler Adult Intelligence Scale, 3rd ed. and Wechsler Memory Scale, 3rd ed. San Antonio: Psychological Corporation (3rd ed.). San Antonio, TX: The Psychological Corporation.

[B109] Wickens C. D. (2008). Situation awareness: Review of Mica Endsley's 1995 articles on situation awareness theory and measurement. Hum. Fact. 50, 397–403. 10.1518/001872008X288420 18689045

[B110] Wickens C. D. Gutzwiller R. Santamaria A. (2015). Discrete task switching in overload: A meta-analyses and a model. Int. J. Hum. Comput. Stud. 79, 79–84. 10.1016/j.ijhcs.2015.01.002

[B111] Wickens C. D. Mavor A. S. McGee J. (1997). Panel on Human Factors in Air Traffic Control Automation (N. A. Press, Ed.). Washington, DC: National Research Council.

[B112] Work J. (2020). Evaluating commercial cyber intelligence activity. Int. J. Intell. Counter Intelligence 33, 278–308. 10.1080/08850607.2019.169087736969684

[B113] Yahya F. Hassanin O. Tariq U. Al-Nashash H. (2020). EEG-Based Semantic Vigilance Level Classification Using Directed Connectivity Patterns and Graph Theory Analysis. World Wide Organisation; IEEE Access.

[B114] Zheng W. L. Gao K. Li G. Liu W. Liu C. Liu J. Q.. (2019). Vigilance estimation using a wearable EOG device in real driving environment. IEEE Trans. Intell. Transport. Syst. 1, 1–15. 10.1109/TITS.2018.2889962

[B115] Zhong C. Yen J. Liu P. Erbacher R. Etoty R. Garneau C. (2015). “ARSCA: A computer tool for tracing the cognitive processes of cyber-attack analysis,” in The 2015 IEEE International Multi-Disciplinary Conference on Cognitive Methods in Situation Awareness and Decision (Xi'an) 10.1109/COGSIMA.2015.7108193

[B116] Zhong S. C. Song Q. F. Cheng X. C. Zhang Y. (2003). “A safe mobile agent system for distributed intrusion detection,” in Proceedings of the 2003 International Conference on Machine Learning and Cybernetics (IEEE Cat. No. 03EX693) (San Diego, CA).

